# Application of mathematical morphology operation with memristor-based computation-in-memory architecture for detecting manufacturing defects

**DOI:** 10.1016/j.fmre.2021.06.020

**Published:** 2021-07-17

**Authors:** Ying Zhou, Bin Gao, Qingtian Zhang, Peng Yao, Yiwen Geng, Xinyi Li, Wen Sun, Meiran Zhao, Yue Xi, Jianshi Tang, He Qian, Huaqiang Wu

**Affiliations:** a School of Integrated Circuits (SIC), Beijing Innovation Center for Future Chips (ICFC), Tsinghua University, Beijing, China; b Beijing National Research Center for Information Science and Technology (BNRist), Tsinghua University, Beijing, China

**Keywords:** Memristor, Computation-in-memory, Mathematical morphology, Defect detection

## Abstract

Mathematical morphology operations are widely used in image processing such as defect analysis in semiconductor manufacturing and medical image analysis. These data-intensive applications have high requirements during hardware implementation that are challenging for conventional hardware platforms such as central processing units (CPUs) and graphics processing units (GPUs). Computation-in-memory (CIM) provides a possible solution for highly efficient morphology operations. In this study, we demonstrate the application of morphology operation with a novel memristor-based auto-detection architecture and demonstrate non-neuromorphic computation on a multi-array-based memristor system. Pixel-by-pixel logic computations with low parallelism are converted to parallel operations using memristors. Moreover, hardware-implemented computer-integrated manufacturing was used to experimentally demonstrate typical defect detection tasks in integrated circuit (IC) manufacturing and medical image analysis. In addition, we developed a new implementation scheme employing a four-layer network to realize small-object detection with high parallelism. The system benchmark based on the hardware measurement results showed significant improvement in the energy efficiency by approximately 358 times and 32 times more than when a CPU and GPU were employed, respectively, exhibiting the advantage of the proposed memristor-based morphology operation.

## 1 Introduction

A vast number of defects, including uneven film surfaces and lithographic patterns, arise during the manufacture of integrated circuits (IC) that may damage an IC and affect its performance. Thus, it is necessary to detect these defects during IC fabrication. However, manual detection of defects requires a large amount of human resources and is prone to human error and leads to low detection accuracy [1]. This has led to the adoption of various automated methods to detect defects during the manufacturing process and save considerable human resources and inspection and correction costs [1, 2]. Specifically, defect detection employing a conventional deep neural network (DNN) can be applied for pattern recognition and classification. In the detection tasks, the defect points to be processed are much smaller, typically containing approximately one to seven pixels, but they are analyzed in an image consisting of 1024 × 1024 pixels or even more [3]. This poses a great challenge for automatic inspection.

Mathematical morphology is an easy to implement and simple to operate method for analyzing and processing geometrical structures and images and is commonly applied during automatic inspection, defect analysis in IC manufacturing, medical imaging analysis, and star point detection [2, 4]. However, this method is also a data-intensive computation technique similar to that of DNN algorithms. Therefore, the use of a central processing unit (CPU) or a graphic processing unit (GPU) are inefficient because of memory wall and power wall issues.

Recently, a computation-in-memory (CIM) architecture was developed to address the memory wall issue. In this architecture, the memory arrays served as computing units and performed traditional data storage functions such as matrix-vector multiplication. Moreover, this architecture reduced the energy consumption and processing latency of the data transfer between the memory and computing units. Several studies have employed CIM based on various memory technologies, including static random-access memory and dynamic random-access memory [5], [6], [7]. Memristor, a two-terminal device, is an emerging non-volatile memory technology with a high operational speed and low energy consumption and is compatible with the traditional CMOS fabrication process [8], [9], [10], [11], [12]. The resistance of a memristor switches between a high resistance state (HRS) and a low resistance state (LRS) when appropriate voltages are applied to the two terminals. The structure of the memristor array is a promising candidate for the hardware implementation of common operations in neural networks or vector-matrix multiplication operations. In the computing process, the conductance of each memristor device corresponds to its weight. The input information is converted to voltage signals with various amplitudes, widths, and pulse numbers and applied to the two terminals of the memristor device. According to Kirchhoff's current law and Ohm's law, the current flow through source lines (SLs) is the product of the input voltage and the conductance matrix [13]. In previous studies, many memristor-based CIM applications have been shown to exhibit advances in high performance and energy efficiency [14], [15], [16], [17], [18], [19], [20], [21], [22], [23]. For example, Cai et al. proposed an integrated memristor–CMOS system that was reconfigured to implement various algorithms. Bilayer PCA and sparse coding were successfully demonstrated on a memristor array [16]. Li et al. implemented a multilayer neural network on a Ta/HfO_2_/Pt memristor array for the MNIST classification task [15]. In 2020, a complete convolutional neural network was fully hardware implemented with multiple memristor arrays [14]. These previous studies focused either on neuromorphic computing applications or simple image processing with a single memristor array.

The implementation of a practical morphology operation on a CIM system faces several challenges. First, traditional morphology algorithms that employ pixel-by-pixel logic computations with very low parallelism [24] cannot be directly implemented on a CIM system that features analog computation and high parallelism. Therefore, a morphology operation with high efficiency and performance is necessary. Second, the goal of the defect detection task is to process small objects in a large image and is different from previous image processing studies. The existing CIM system [25] for convolutional neural networks (CNNs) cannot be used for morphological operations. Thus, the development of a new architecture and implementation scheme is required. Finally, in previous applications, the defects in images were smaller than the patterns to be processed. The construction of a CIM system for morphological operations requires memory devices with excellent reliability.

Morphology operations have not been demonstrated on a practical CIM system because of the abovementioned challenges and limitations. In this study, for the first time, we proposed a highly efficient and accurate implementation scheme for memristor-based morphology operations. We designed a complete CIM architecture and implemented a detection process using a 16-Kb multi-array hardware system [14]. Defect detection in IC manufacturing images and medical images were experimentally demonstrated using the proposed memristor-based CIM morphology operation system. The benchmark results for the system showed significant improvements in speed and energy efficiency compared to that of a CPU or GPU. This is the first non-neuromorphic-computation demonstration of a multi-array-based memristor system.

## 2 Morphology operation and architecture design

**Fig. 1:**
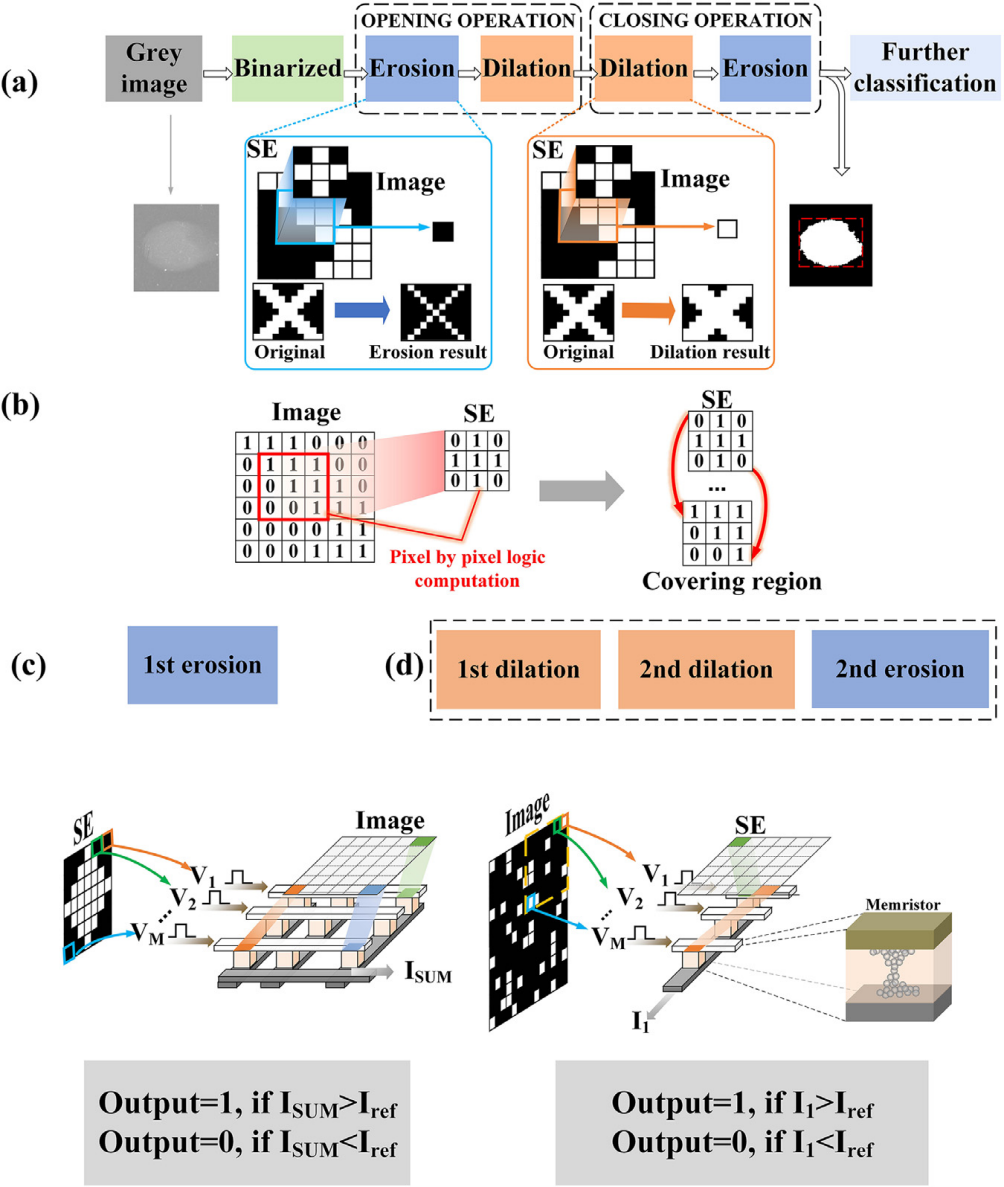
**Principles of morphology operations and hardware implementation schemes.** (a) flowchart of morphology operations for defect detection, including binarization and opening and closing operations. Erosion first, then dilation operation, is called an opening operation. Conversely, dilation first, then erosion operation, is called a closing operation. (b) Implementation scheme of morphology operations. The pixel-by-pixel logic computation for binary morphology operation with very low parallelism is converted into two steps suitable for hardware implementation in memristor arrays. Step 1: calculate the product and accumulation results of the SE and corresponding image window. Step 2: compare the results of Step 1 and the critical conditions, that is, the final dilation or erosion result. (c–d) Diagram of memristor-based morphology operations. (c) First erosion layer. The image is mapped to the memristor array and the erosion SE is converted to read voltage pulses and fed to the corresponding BLs. The eroded value is the comparison result between the collected current flow through SL and the reference value. (d) Second erosion layer and first and second dilation operations. The dilation or erosion SE is mapped to the memristor arrays. The operation results of the previous layer are transferred to multiple voltage pulses and applied on corresponding BLs. The current flow through SL is compared with I_ref_.

The basic morphological operations include dilation, erosion, opening, and closing. In this study, we implemented binary morphology. Both dilation and erosion operations involve the convolution of an image and structuring elements (SEs), including disk, line, and diamond-shaped structures. The pixels in a binary SE and image are “0” or “1,” respectively. For example, in the dilation process, we assumed that the SE contained 3 × 3 binary pixels (five white and four black pixels) as shown in Fig. 1(a). According to Eq (1), the goal of the dilation operation is to find the local maximum value in an image region assigned with a “1” that contains two black and three white pixels. In addition, the currently processed pixel was aligned with the center pixel of the SE of the dilation operation when it was located in the third row and third column of the image. The maximum value of an SE with a corresponding area of “1” was assigned to the current operating pixel. Then, the SE of the dilation moved to the right or down. The morphology operation of the next pixel was performed in a similar manner. Conversely, in an image area covered by “1” in the SE during the erosion operation, “0” was assigned to the eroded pixel if one pixel was found to be zero. The process of the erosion operation was equivalent to finding the local minimum value in the corresponding window covered by “1” in the SE as presented in Eq. (2). The two operations, dilation and erosion, are often performed together because they significantly affect the area of the pattern and realize more complex operations. The opening operation removes isolated noise pixels and smooths image contours and consists of both erosion and dilation operations. However, the closing operation or the converse operation order is suitable for filling small holes in the connected region and bridging narrow gaps (see Eqs. (3) and (4)).

(1) $$\text{Dilation}:\;\;\;A⊕B=\left\{x|\left(B_{x}\right)\cap A\neq \emptyset \right\}$$

(2) $$\text{Erosion}:\;\;\;A⊖B=\left\{x|\left(B_{x}\right)\subseteq A\right\}$$

(3) Opening:(A⊖B)⊕C

(4) Closing:(A⊕B)⊖C

A flowchart of the detection process is shown in Fig. 1(a). Initially, the gray image was binarized, and the shapes of the defects were apparent. Moreover, these defects were transformed into a clear pattern using successive opening and closing operations. In this study, we proposed a memristor-based morphology operation scheme (Fig. 1(c)–(d)) to address the identified challenges and limitations of the implementation of this system. Considering the above principle of dilation and erosion operations, the pixel-by-pixel logic computations were converted in comparison with the accumulation result and that in the critical condition as illustrated in Fig. 1(b). In this study, we employed two diamond SEs composed of nine (first erosion and second dilation) and 25 binary pixels (first dilation and second erosion). The reference values for erosion and dilation were different.

The images or SEs were mapped to the memristor arrays and fed to the horizontal terminals of the memristor array. A comparison of the total current flow through the vertical terminals, I_sum_, and the I_sum_ in the critical condition, I_ref_, obtained the final dilation or erosion results. The reference value was related to the SE and the type of operation as shown in Eqs. (5) and (6). I_1_ and I_2_ are the currents when the memristor cell was programmed to LRS and HRS, respectively, while σ_1_ and σ_2_ represent the sensing tolerance. We assumed that the SE contained *x* “1” pixels and *y* “0” pixels. The input fed to the bit lines (BLs) was the result of the previous layer. Under critical conditions, the current values were I_1_ and I_2_ × *y*, respectively, indicating the necessity to have a current reference greater than I_2_ × *y* and less than I_1_. An analog-to-digital converter (ADC) was used to detect the current flow through the SL and compare it with the reference value because the input of the first erosion SE required multiple cycles. The following dilation and erosion comparison results were implemented with a sensing amplifier (SA).

(5) $$\text{Erosion}:\;\;I_{\text{ref}}=\left(x-1\right)\times I_{1}+\sigma_{1}$$

(6) $$\text{Dilation}:\;I_{\text{ref}}=1\times I_{1}+\sigma_{2}$$

**Fig. 2:**
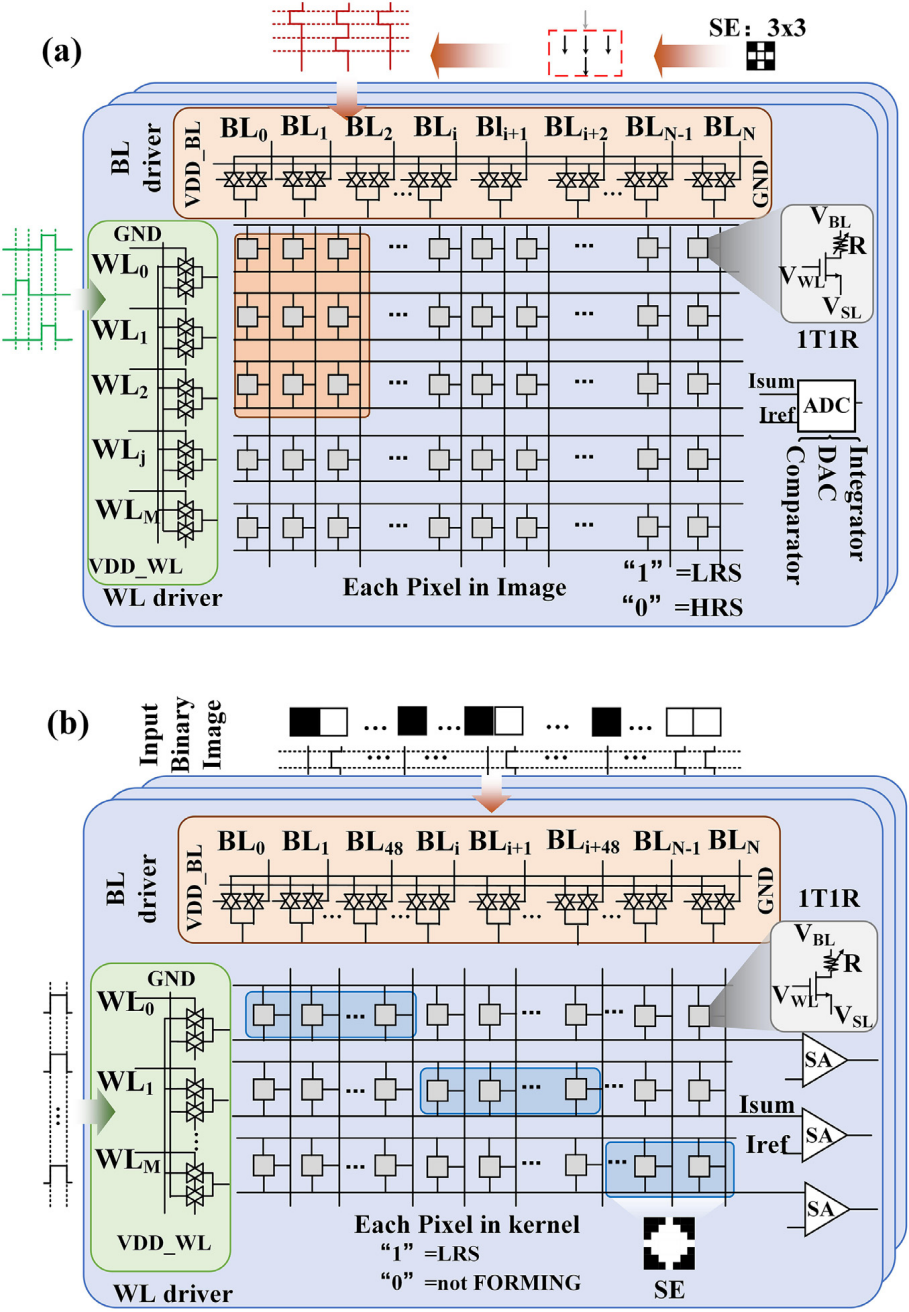
**Schematic diagram of four-layer memristor-based morphology operations.** (a) Circuit schematic of first erosion operation in memristor-based morphology operation system. It contains memristor array, BL driver, WL driver, and ADC (DAC, comparator, and integrator). In the first erosion operation, the defect image is segmented and stored in the memristor array. Each memristor cell corresponds to a pixel in the image. The pixels in the erosion SE are converted to read voltage pulses (“1” and “0” in the SE, respectively, correspond to 0.2 V and 0 V) and fed to the BLs corresponding to the covered image window. WLs are opened line-by-line according to the scheme on this figure. The comparison results between the reference value and the collected current were implemented by connected ADC and fed to the next morphology layer as input values. (b) Circuit schematic of first and second dilation and second erosion operation in the memristor-based morphology operation system. Taking an SE with 5 × 5 binary pixels as an example, to implement high parallel morphology operations, the erosion or dilation SEs are replicated and mapped into multiple rows of memristor arrays. It is different from the first layer. The morphology outputs of the previous layer are converted to voltage pulses (“1” corresponds to 0.2 V, “0” corresponds to 0 V). These pulses are fed in the corresponding BLs while all WLs are open. Each SL is connected with SA for comparison.

We designed a high-efficiency memristor-based auto-detection system architecture consisting of four morphology layers as shown in Fig. 2. In the first erosion operation, the binarized defect image was segmented and mapped into multiple memristor arrays as the first layer, which was different from the operations in existing neuromorphic computation applications. In addition, a “1” in the binary erosion SE denoted a read pulse (0.2 V), and a “0” denoted no pulse (0 V). The voltage pulses were fed into the BLs corresponding to the image window. At the same time, the corresponding word line (WL) of the memristor array was open row by row. Considering that the utilized SEs were symmetrical, the process of inputting the SE into the memristor arrays can be simplified. The total current of the corresponding source lines (SLs) was compared with the reference value. The outputs were fed to the next layer for other morphological operations. The goal of the proposed scheme for the remaining three layers was to map the erosion or dilation of the SEs into multiple rows of memristor arrays. The input data were the operated images from the previous layers. The pixels were converted to read pulses (“1” and “0” pixels are converted to 0.2 and 0 V, respectively) and fed to the BLs corresponding to the pixels in the SEs (Fig. 1(d) and Fig. 2(b)). In addition, an appropriate voltage was applied to all WLs in the memristor array to ensure that all the transistors were open. The current flow through the SLs was sent to the connected SA for comparison. Thus, multiple pixels were operated in parallel.

## 3 Memristor array and hardware implementation system

**Fig. 3:**
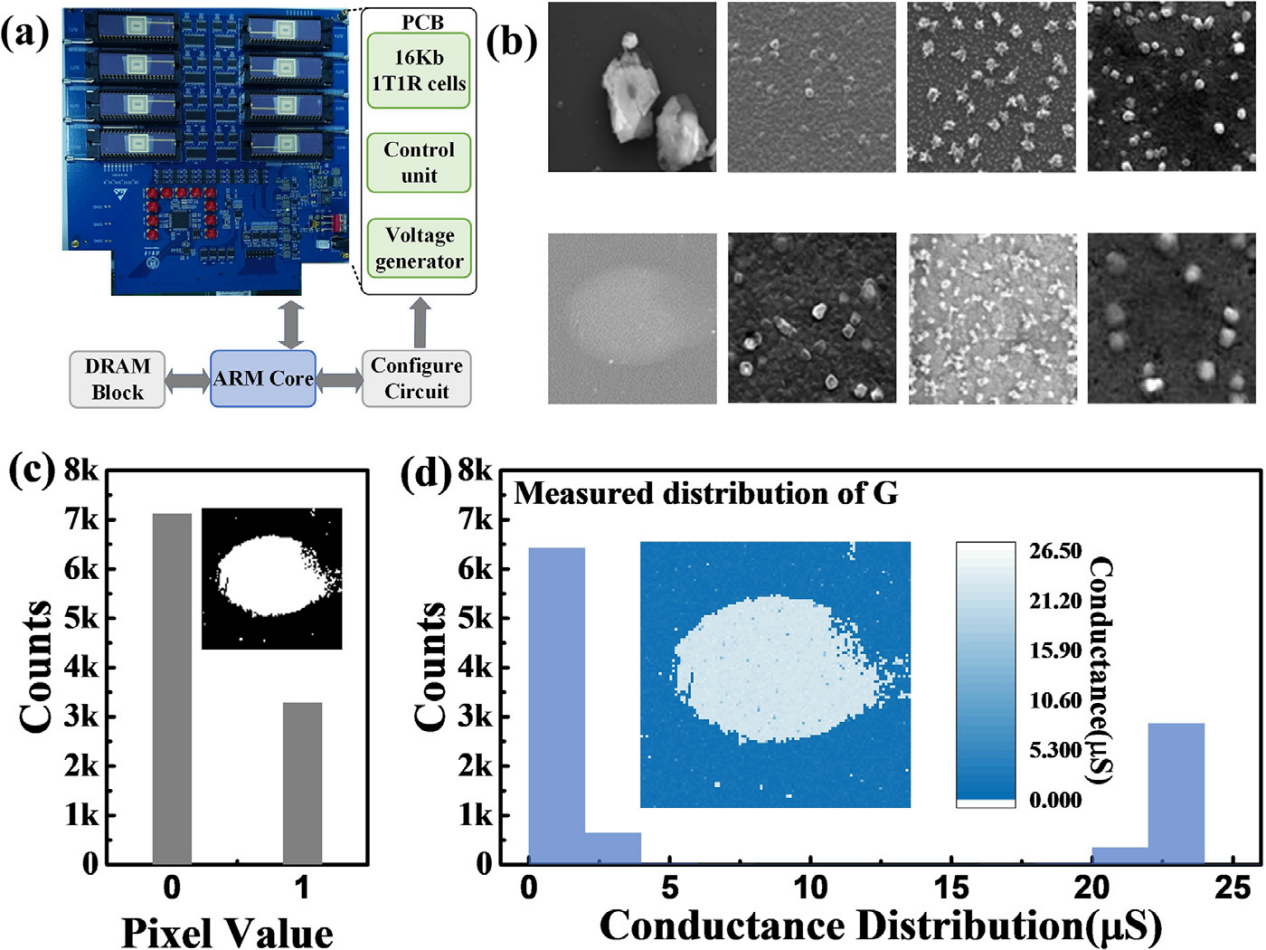
**Hardware system and experiment for defect detection.** (a) Measurement platform including FPGA board and PCB containing 16-Kb 1T1R memristor cells, and control unit, (b) eight original defect images in semiconductor manufacturing process. Each image contains 100 × 100 pixels, (c) original binarized defect image and value distribution of the image pixels (black and white pixels respectively correspond to “0” and “1”), and (d) measured conductance distribution of defect image after programming process in first erosion layer.

We fabricated a fully hardware-implemented CIM system consisting of 16-Kb memristor cells, eight 2-Kb arrays, and a voltage generator and control unit to verify the feasibility of the proposed architecture and morphology operation scheme as shown in Fig. 3(a) [14]. An FPGA board was connected to a printed circuit board (PCB) for data input and control. The integrated 2-Kb memristor array containing 128 rows and 16 columns was fabricated using 130-nm technology. In this study, we used a 1T1R memristor with a TiN/TaO_x_/HfO_x_/TiN stack with an HfO_x_ switching layer, a TaO_x_ layer as the thermal enhanced layer (TEL), and TiN layers as electrodes. The transistors were processed by the standard CMOS foundry process, and the memristor devices were fabricated in the laboratory. The TiN layers were sequentially deposited on the received wafer. The WL of the transistor was connected to the memristor cell for current limitations and selection. The conductive filament was formed in the switching layer, and the resistance of the memristor decreased when an appropriate voltage was applied to the electrodes.

**Fig. 4:**
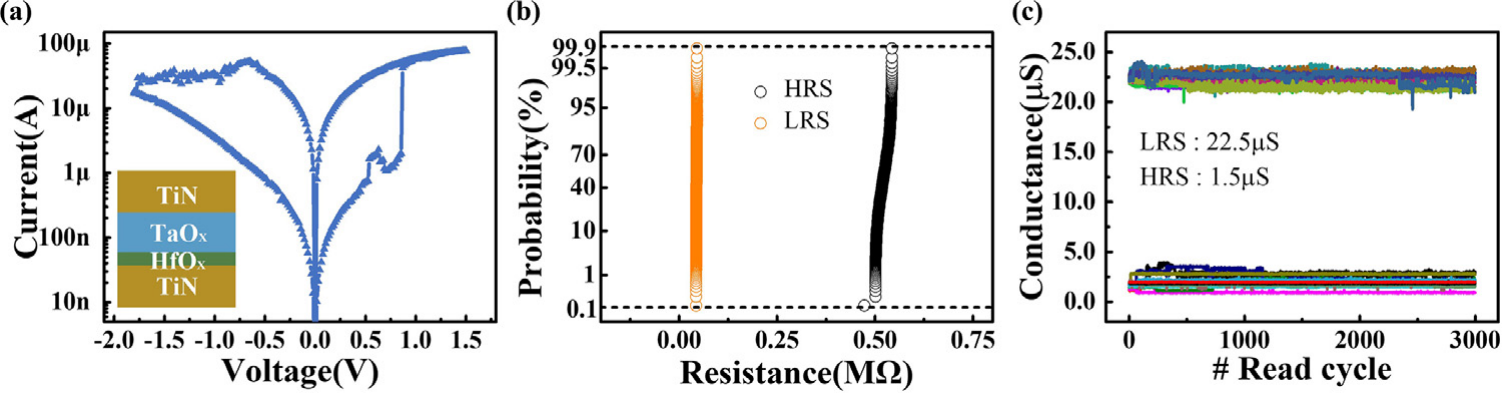
**Basic device characteristics of memristor arrays.** (a) Typical I-V curve of TiN/TaO_x_/HfO_x_/TiN-based memristor cell. The embedded figure is the device stack. The TiN layers act as device electrodes. TaO_x_ works as a thermal enhanced layer (TEL). The conductive filament (CF) is formed in the HfO_x_ layer during the switching process. (b) Cell-to-cell resistance distribution of HRS (1.5μS) and LRS (22.5μS) in the memristor array. (c) Conductance fluctuation of memristor cells during 3000 read cycles (read pulses: 0.2 V).

**Fig. 5:**
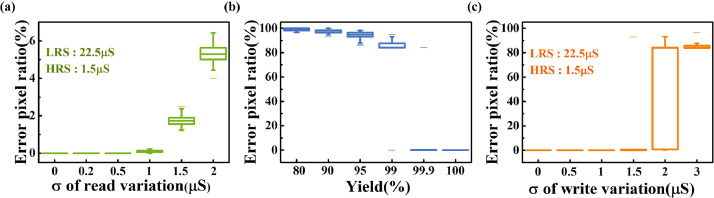
**Simulation results of defect detection under various non-ideal factors.** (a) Pixel error ratio of detection region under various read variations for memristor-based morphology operations. (b) Pixel error ratio of detection region under various yield values for memristor-based morphology operations. The yield is defined as the proportion of memristor devices that cannot perform the forming operation. (c) Pixel error ratio of detection region under various write variations during programming process for memristor-based morphology operations.

We propose a new method for implementing morphology operations in memristor arrays. However, the non-ideal characteristics of the memristor device (Fig. 4), such as read and write noise and variation, affected the detection accuracy. A high uniformity of HRS and LRS (HRS = 22.5 μS and LRS = 1.5 μS) was required to ensure efficient detection performance. A memristor that could not perform the forming operation was considered a failure device in the array and also affected the defect detection. In addition, it was necessary to ensure that the fluctuations of the stored data in the computing process were within a certain range to obtain a satisfactory result. The obscure defect pattern in the first defect image (Fig. 3(b)) was apparent. In the simulation, large non-ideal factors caused performance degradation. We simulated the area of the extracted defect region under various amplitudes of read noise, programming variations, and yield to quantify the effects of these non-ideal factors. Fig. 5(a)–(c) show the simulated error pixel ratio defined in Eq. (7) under various non-ideal factors. From the equation, we defined N_non-ideal_ as the total number of white pixels in the extracted defect pattern under various non-ideal factors and N_ideal_ as the number of white pixels in the defect pattern by ideal morphology operations.

(7) $$R_{\text{Simulation}}=\left(N_{\text{non}-\text{ideal}}-N_{\text{ideal}}\right)/N_{\text{ideal}}$$

The memristor-based morphology operations have a certain tolerance to non-ideal device characteristics based on the simulation results. Some significant cells may be damaged when these non-ideal factors are large and result in unsatisfactory outputs. Therefore, these factors should be controlled within a much smaller range than a typical neuromorphic computation application to ensure the correct operation of the CIM system. For example, the yield of the memristor array has to be greater than 99.9%, and the standard deviations of the read and write processes should be less than 1 and 1.5 μS, respectively.

The typical I-V curves of the memristor device are shown in Fig. 4(a). The memristor cells in the array having multiple voltages and verified pulses were programmed using the HRS or LRS. Fig. 4(b) shows that the HRS and LRS distributions of the integrated memristor array had excellent cell-to-cell uniformity. The conductance states of the memristor cells were recorded after being programmed for 3000 read cycles. The amplitude of the read voltage pulse was 0.2 V to avoid switching from HRS to LRS or LRS to HRS in the memristor cells. Fig. 4(c) presents the conductance fluctuations of the programmed memristor cells. The conductance of memristor cells was maintained in a stable state during multiple read cycles.

## 4 Results of morphology operation

We first demonstrated the defect detection of the generated SEM images of the IC manufacturing process to verify the feasibility of morphology operations in the memristor array. In the experiments, we employed eight SEM images as test samples as shown in Fig. 3(b), wherein each defect image contained 100 × 100 pixels. For the defect detection task, the overall processing architecture contained four CIM morphology operation layers, including two dilation and two erosion operations.

**Fig. 6:**
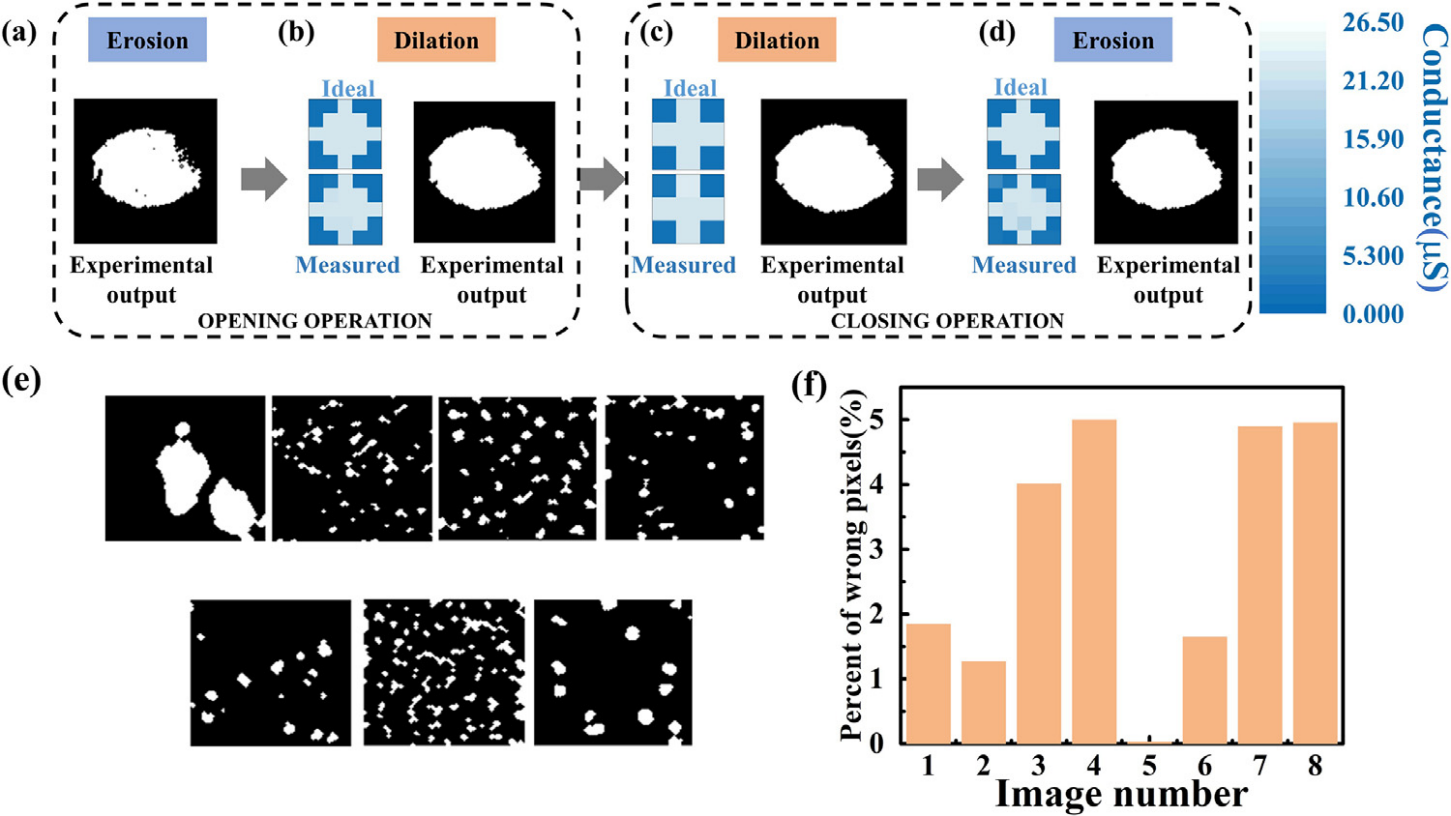
**Experimental four-layer morphology operation results of defect image:** (a) experimental eroded image in first erosion layer, (b) first dilated image result and ideal and actual measured conductance distribution of dilation of SE, (c) image result of second dilation layer and ideal and experimental measured conductance distribution of SE, (d) experimental output of second erosion layer and ideal and experimental measured conductance distribution of SE, (e) experimental morphology operation results of seven other defect images with two dilation and two erosion operations, and (f) pixel error ratio in demonstration results of eight defect images.

The first defect image showed an obscure oval-shaped pattern at the center of the image as shown in Fig. 3(b). First, the captured gray defect image was converted into a binary image and stored in the memristor arrays using multiple programming and verification pulses. The memristor devices corresponding to the white pixels were programmed to LRS (22.5 μS), while the black pixels were programmed to HRS (1.5 μS). Fig. 3(c) shows the value distribution of the binarized image and a statistical graph of the ideal conductance distribution where most pixels in the image were black. Moreover, the conductance distribution of the defect image after programming was close to the ideal conductance distribution as shown in Fig. 3(d). In the first erosion operation, “1” pixels in the first row of the binary erosion SE were converted into pulses (0.2 V) which were then fed into the BL-covered region by the erosion of the SE. Then, the voltage pulses corresponding to the pixels in the second row were fed to the same BLs. The process continued until all binary pixels in the SE were converted and sent to the BLs. In addition, the WLs of the memristor array corresponding to the covered region were opened row by row. Moreover, the total current flow of the corresponding SLs was collected and compared with I_ref_ after the input process of the erosion SE was conducted. This erosion operation was repeated until all pixels in the defect image were morphologically processed. Fig. 6(a) shows the experimentally eroded outputs in the first erosion layer. The area with the highest noise in the figure was removed in the first erosion layer because it was relatively smaller than the SE. However, some holes were generated inside the defect pattern, indicating the necessity for further processing. The results in the first erosion layer were sent to the next morphology operation layer. Furthermore, all pixels in the dilation or erosion SEs in the second, third, and fourth morphology operations were mapped into multiple memristor arrays using multiple programming and verified pulses, where HRS was used to program the black pixels in the SE, and LRS was employed to map the white pixels. As illustrated in Fig. 6(b)–(d), the conductance distribution of the measured erosion or dilation SEs was found to be similar to that of the ideal SEs. The corresponding image window was converted to voltage pulses and sent to the corresponding BLs during the dilation or erosion operations of these three layers. The current flow through the SL was collected and compared. Specifically, the morphologically operated pixel was assigned as “1,” otherwise as “0” if the current was greater than I_ref_. Then, the output results were fed to the next layer until the four-layer morphology operations were completed. Fig. 6(b)–(d) show the experimental outputs in the first, second, and second erosion layers, respectively. The shape of the defect found in the middle of the image was clear and was used for further analysis, verifying the feasibility of the four-layer memristor-based morphology operation. Fig. 6(e) shows the results of the experiments conducted on the other seven defect images by morphology processing. The four-layer CIM-based system successfully extracted defects and achieved a satisfactory morphology operation function verified through the comparison of the original SEM images and experimental results (Fig. 3(b) and Fig. 6(e)).

We calculated the pixel error ratio of eight test images to evaluate the memristor-based morphology processing effect for different defect images as shown in Eq. (8). In the equation, N_hardware_ is the total number of white pixels in the extracted defect processed by the hardware, and N_software_ is the ideal number of white pixels of the morphology operation output by the software. As presented in Fig. 6(f), the pixel error ratio of most defect images was less than 5% and was calculated as

(8) $$R_{\mathrm{Eval}\mathrm{uati}\mathrm{on}}=\left(N_{\mathrm{hard}\mathrm{ware}}-N_{\mathrm{soft}\mathrm{ware}}\right)/N_{\mathrm{soft}\mathrm{ware}}$$

**Fig. 7:**
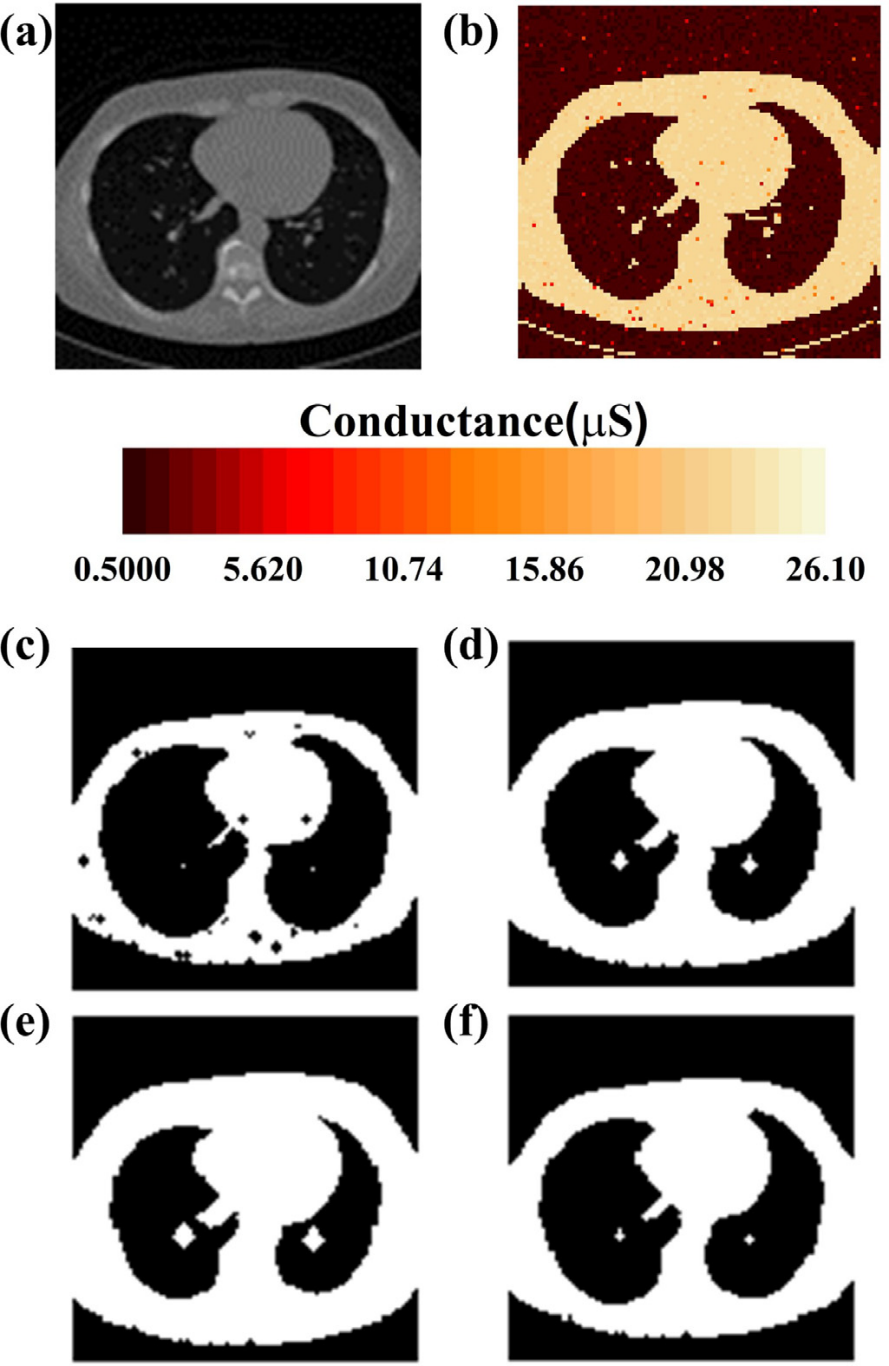
**Experimental four-layer morphology operation results of lung image:** (a) original lung image from LUNG16 dataset, (b) actual measured conductance distribution after programming process of lung image. Experimental morphology operation results of lung image: (c) first erosion layer, (d) first dilation layer, (e) second dilation layer, and (f) second erosion layer.

In addition, mathematical morphology is often used for defect detection tasks in the medical image processing such as tumor region segmentation and abnormal brain image detection [26]. Furthermore, the opening and closing operations were used to smooth the boundary contours and fill the holes inside for further analysis. This ensured that the size of the processed area remained unchanged. Moreover, the four-layer memristor-based morphology operation can also be used in medical image processing. We experimentally demonstrated the extraction of the shape of a lung image from the LUNG16 dataset [27], wherein the morphology operation processing of a lung image was composed of two dilation and two erosion operations. Fig. 7(a) shows the original lung image. The gray lung image was binarized and mapped to memristor arrays. Fig. 7(b) illustrates the conductance distribution of the memristor arrays after the mapping process. The experimental outputs of the four morphology layers are shown in Fig. 7(c)–(f). With successive opening and closing operations, the memristor-based morphology operation obtained a good medical image processing result for further analysis. The SEs used were the same as those used in the defect detection tasks.

**Fig. 8:**
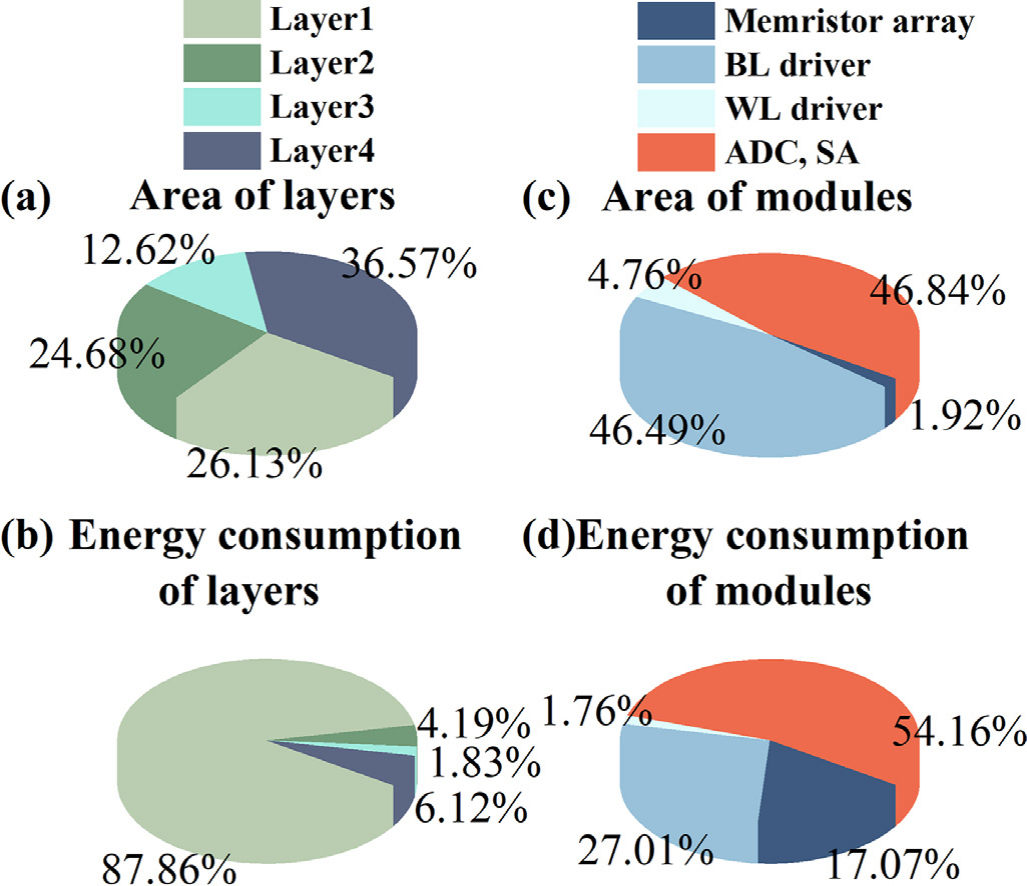
**Benchmark results of four-layer morphology system in 130-nm technology.** Each morphology operation layer was composed of 1T1R memristor arrays, BL driver, WL driver, ADC, or SA. The data of ADC and SA were actual circuit test results. The other circuit modules were simulated with simulator XPEsim. (a) Occupied area breakdown of four morphology layers, (b) energy consumption breakdown of four morphology layers, (c) occupied area breakdown of different circuit modules, and (d) energy consumption breakdown of different modules.

Furthermore, we benchmarked the four-layer CIM-based morphology operation system in which each layer consisted of BL and SL drivers, 1T1R memristor arrays, and SA (or ADC). The data of the SA module were obtained from an actual circuit test. Meanwhile, the other peripheral circuits were simulated using XPEsim [28, 29]. The overall system exhibited a pipeline structure, and the first and fourth layers occupied a relatively large area as shown in Fig. 8(a). However, the first layer consumed the most energy (Fig. 8(b)). As shown in Fig. 8(c)–(d), the ADC and SA occupied most of the area and consumed the most energy, while the memristor arrays only occupied 1.92% of the total area and consumed 17.07% of the energy. The final area, latency, and energy consumption of this four-layer system exhibited an energy efficiency of 3,209 GOP s^−1^W^−1^ and are listed in Table 1. As illustrated in Table 2, we compared the Intel E5-2699 v3 processor and the Nvidia Tesla V100. The computing energy efficiency of the CPU was 8.96 GOP s^−1^W^−1^, and that of the GPU was 100 GOP s^−1^W^−1^ [30, 31]. The proposed memristor-based morphology operation system offered a higher energy efficiency for defect detection tasks and showed great potential for automatic image processing.

**Table 1:** **Final performance evaluation results of four-layer memristor-based morphology operation system in 130-nm technology (final benchmark results include power, area, and hardware performance)**.

Parameter (130nm technology)	Result
Power (mW)	13
Performance (GOP s^−1^)	42
Area (mm^2^)	17

**Table 2:** **Energy efficiency of four-layer memristor-based morphology system and comparisons results with common hardware platforms**.

Hardware	Energy efficiency (GOP s^−1^W^−1^)
CPU (Intel E5-2699 v3) [10]	8.96
GPU (NVidia Tesla V100) [11]	100
CIM system (130nm technology)	3,209

## 5 Conclusion

In this study, we proposed a novel architecture for a memristor-based morphology operation method with high parallelism for defect detection. Moreover, we demonstrated the excellent reliability of memristor arrays to ensure the accuracy of detection. For the multi-array CIM system that was completely hardware implemented, we experimentally demonstrated the detection of defects during IC manufacturing and for the image extraction of lungs. Moreover, the benchmark results of the proposed memristor-based CIM morphology operation system showed advantages in terms of processing speed and energy efficiency compared to traditional methods. In addition, the memristor-based CIM system performed operations other than vector-matrix multiplication and completed more complex tasks. Finally, it has the potential to become a more flexible and versatile integrated CIM system with high performance.

## Declaration of Competing Interest

The authors declared that they have no conflict of interest to this work.
